# Proteomic phenotyping of metastatic melanoma reveals putative signatures of MEK inhibitor response and prognosis

**DOI:** 10.1038/s41416-018-0227-2

**Published:** 2018-08-17

**Authors:** Christoph Krisp, Robert Parker, Dana Pascovici, Nicholas K. Hayward, James S. Wilmott, John F. Thompson, Graham J. Mann, Georgina V. Long, Richard A. Scolyer, Mark P. Molloy

**Affiliations:** 1 Australian Proteome Analysis Facility (APAF), Department of Chemistry & Biomolecular Sciences, Macquarie University, Sydney, NSW Australia; 2 University Medical Center Hamburg, Institute for Clinical Chemistry and Laboratory Medicine, Mass Spectrometric Proteomics Group, Hamburg, Germany; 3 Oncogenomics Laboratory, QIMR Berghofer Medical Research Institute, Brisbane, QLD Australia; 4 Melanoma Institute Australia, The University of Sydney, Sydney, NSW Australia; 5 Royal Prince Alfred Hospital, Sydney, NSW Australia; 6 Royal North Shore Hospital, Sydney, NSW Australia; 7 Kolling Institute, The University of Sydney, Sydney, NSW Australia

## Abstract

**Background:**

Genotyping of melanomas is used to identify patients for treatment with BRAF and MEK inhibitors, but clinical responses are highly variable. This study investigated the utility of protein expression phenotyping to provide an integrated assessment of gene expression programs in *BRAF/NRAS* melanoma which would be useful for prognosis and may predict response to MEK inhibition.

**Methods:**

Mass spectrometry profiling of early passage cell lines established from Stage III cutaneous melanomas was conducted. Basal protein expression was correlated with in vitro response to the MEK inhibitor, selumetinib. Protein expression in a cohort of 32 drug naïve *BRAF/NRAS* metastatic melanoma specimens was examined. The prognostic utility of a subset of these proteins and mRNA transcripts from a separate cohort was determined.

**Results:**

Unsupervised analysis of basal cell line protein abundances delineated response to selumetinib, but *BRAF/NRAS* genotype did not. Resistance was associated with functions including cell motility, cell adhesion and cytoskeletal organization. Several of these response biomarkers were observed in lymph node biospecimens and correlated with melanoma-specific survival. Loss of ICAM-1 protein and mRNA expression was a strong prognosticator of diminished survival in *BRAF/NRAS* mutant melanoma.

**Conclusions:**

These results demonstrate the utility of proteomic phenotyping to identify both putative biomarkers of response to MEK inhibition and prognostication associated with metastatic melanoma.

## Introduction

In cutaneous melanoma, mutations of either the Ser/Thr protein kinase BRAF (approximately 40%), or the small GTPase NRAS (approximately 25%) are most common.^1,2^ Mutations in either of these proteins are usually mutually exclusive and result in unrestrained activation of the MAPK cell proliferative pathway. In clinical practice, it is now routine to screen for BRAF V600 mutations in metastatic melanoma patients^3,4^ since there are approved targeted therapies to treat advanced melanoma patients. However, in spite of this genotypic information, patient response greatly varies.^5,6^ Approximately 5–10% of patients do not respond as expected due to innate resistance, whilst the responses in the remaining patients vary significantly.^7–9^ For example, in an open-label randomized, phase 3 study of 704 patients with metastatic melanoma comparing dabrafenib plus trametinib versus vemurafenib monotherapy,^8^ the objective response rate was 64% (95% CI 59–69) in the combination-therapy group versus 51% (95% CI 46–57) in the vemurafenib group. Early identification of long-term responders to targeted therapy is an unmet clinical need.^10^

In recent years, research has focused on identifying the mechanisms by which melanomas transition from a drug sensitive to a resistant phenotype.^11^ These processes arise directly as a result of selective pressures where tumors reoccur with cell populations enriched for further genetic aberrations^12–14^ and/or have transitioned to utilize alternative signaling processes that can re-establish MAPK activity, cell survival and proliferation.^15,16^ The complexity of the responses observed in patients during therapy is indicative of tumor plasticity, and reflects a heterogeneous disease where drug sensitivity may depend on several intrinsic factors (tumor stage, intra-tumor heterogeneity, tumor microenvironment, and individual genetic and epigenetic diversity).^17^ In the tumor microenvironment stromal cells can secrete hepatocyte growth factor (HGF) that leads to MAPK reactivation by signaling through the receptor tyrosine kinase MET.^18,19^ Broad transcriptional programs are active in melanocyte development and pigmentation and are associated with proliferative or invasive phenotypes. These have also recently been correlated with MAPK dependency and response to therapy in vitro.^20–22^

Here, we explored the use of protein phenotyping by SWATH mass spectrometry^23^ to provide a readout of MAPK pathway activity in *BRAF/NRAS* mutated melanoma cells. We hypothesized that the basal cancer proteome reflects an integrated signal of gene expression programs and this may have superior utility for predicting response to MEK inhibition than the current clinical standard of targeted genotyping. We validated our findings in fresh-frozen lymph node metastatic melanoma specimens, and further, determined prognostic associations with melanoma specific survival (MSS) based on a three-protein biomarker signature. Our results demonstrate the feasibility of protein-based screening of tumors to identify likely response to targeted drug treatment, which could not be predicted *a priori* by the current clinical standard of targeted mutational genotyping.

## Materials and methods

### Cell culture

Stage III early passage melanoma cell lines C002, C037, C045, C052, C054, C078, C084, and C096 were generated from fresh tissue melanoma specimens from patients with melanomas of cutaneous origin that presented with superficial spreading or nodular melanoma subtypes. D22M was established from a Stage IV patient (Supplementary Table 1). The mutational status was assessed using a melanoma-specific panel.^24^ The cell lines were cultured in 10% (v/v) bovine serum supplemented RPMI 1640 medium (Life Technologies) at 37 °C in a 5% CO_2_ atmosphere. Cell lines were harvested at 80% confluence.

### Cell viability assay

Cell lines were seeded in 96-well plates at 5000 cells/well in triplicate for each drug treatment. After 2 h adherence cells were treated with serial dilutions (10, 2, 1, 0.2, and 0.02) of MEK inhibitor selumetinib (AZD6244; Selleckchem) dissolved in DMSO. After 10 days of growth the viability for each cell line was assessed by Presto Blue Assay and compared to DMSO only treated cells (Life Technologies).

### Melanoma tissue

The fresh-frozen melanoma tumor samples were obtained from the Melanoma Institute Australia Biospecimen Bank, accrued with written informed patient consent and approved by Institutional Review Board (Sydney South West Area Health Service institutional ethics review committee (Royal Prince Alfred Hospital (RPAH) Zone) Protocol No. X08-0155/HREC 08/RPAH/262, No. X11-0023/HREC 11/RPAH/32, and No. X07-0202/HREC/07/RPAH/30). Twenty-six tissue specimens were taken from regional lymph nodes of AJCC Stage III patients, 3 specimens were from regional lymph nodes of AJCC Stage IV patients and a further 3 specimens were from distant lymph nodes of AJCC Stage IV patients. In total 32 tumors were studied; 16 NRAS mutant and 16 BRAF mutant melanomas with patient follow-up exceeding 10 years (Supplementary Table 2). Melanoma-specific survival was calculated from the date of resection. None of the patients were treated with BRAF or MEK inhibitor targeted therapies or immune check point inhibitors therapy. The most common treatments were adjuvant radiotherapy or experimental vaccinations. Amongst these specimens, 6 NRAS and 7 BRAF patients showed poor survival after sample collection (≤2 years), whereas the other 9 NRAS and 7 BRAF patients showed good survival (>4 years).

### Protein preparation and digestion

Cultured cells were lysed and proteins denatured in 100 mM triethyl ammonium bicarbonate (TEAB, Sigma Aldrich) and 1% sodium deoxycholate (SDC, Sigma Aldrich) buffer (pH 7.8) for 5 min at 99 °C. After lysis Benzonase ® nuclease (Sigma Aldrich) was added and incubated for 30 min at room temperature to degrade DNA (1:10,000 enzyme/DNA). Tissue samples were lysed in 100 mM TEAB and 1% SDC buffer with 10 pulses of a probe sonicator and boiled for 5 min at 99 °C. Protein concentration were estimated using the bicinchoninic acid protein assay (Pierce). For the cell lines, cysteine residues were reduced in presence of 10 mM dithiothreitol (DTT, Bio-Rad) at 60 °C and alkylated with 10 mM iodoacetamide (IAA, Bio-Rad) at room temperature in the dark. Trypsin (Promega, sequencing grade) was added in a 1:50 ration and proteins were enzymatically degraded overnight at 37 °C. By adding 1 µL formic acid (FA, Ajax Finchem) the digestion was quenched and the SDC precipitated and removed by centrifugation (14.000 rpm) for 5 min. Samples were lyophilized and reconstituted in 2% acetonitrile (ACN, Merck) and 0.1% FA. The tissue samples (20 µg) were mixed with the equivalent amount of 4× NuPAGE loading buffer (Thermo Fisher Scientific) with DTT, were loaded onto a 4–12% polyacrylamide gel (Thermo Fisher Scientific) and run for 1 cm into the gel. Gels were stained for an hour in Coomassie blue G-250. Entire 1 cm band for each sample was cut in to small cubes and transferred into a 1.5 mL tube, destained, again reduced with 10 mM DTT, alkylated with 20 mM IAA and digested with trypsin (1:20 ration) over night. Peptides were eluted from the gel pieces with 80% ACN and 0.1% FA, lyophilized and re suspended in reconstituted in 2% ACN and 0.1% FA.

### LC-MS/MS

LC-MS/MS analyses of cell line samples were carried out using a NanoLC^TM^ ultra with cHiPLC® system (SCIEX). For RP LC-MS/MS, 200 µm x 0.5 mm nano cHiPLC trap column (ChromXP^TM^ C18-CL 3 µm 120 Å; SCIEX) and 15 cm × 75 µm nano cHiPLC columns (ChromXP^TM^ C18-CL 3 μm 120 Å) were used. Multiphase LC-MS/MS using a prototype multiphase trap chip was conducted as described previously^22^ using a TripleTOF 5600 mass spectrometer (SCIEX) and 80 min acetonitrile gradients.

LC-MS/MS analysis for tissue samples were performed on an Ekspert NanoLC 400 with cHiPLC system (SCIEX) coupled to a TripleTOF 6600 mass spectrometer (SCIEX). In RP LC-MS/MS mode, a 200 µm x 0.5 mm nano cHiPLC trap column and 15 cm × 200 µm nano cHiPLC columns (ChromXP^TM^ C18-CL 3 μm 120 Å) was used with 140 min ACN gradients.

For data dependent MS/MS acquisition, 20 most intense *m*/*z* values exciding a threshold >150 counts per second on the TripleTOF 5600 (cps) and 250 cps on the TripelTOF 6600 with charge stages between 2+ and 4+ were selected for analysis from a full MS survey scan and excluded form analysis for 20 s to minimize redundant precursor sampling.

In data independent acquisition *m*/*z* windows of 12.5 Da were used over a range of 400–1250 *m*/*z* on the TripleTOF 5600 and a 100 variable window method over a range of 400–1250 *m*/*z* on the TripleTOF 6600 with window sizes based on precursor densities in the LC-MS/MS acquisition. Collision energies were calculated for 2+ precursors with *m*/*z* values of lowest *m*/*z* in window +10% of the window width. The data were acquired over an 80 min ACN gradient.

Selected reaction monitoring (SRM) of peptides selected from SWATH-MS acquisitions were carried out on a QTRAP® 5500 (SCIEX) with NanoAcquity UPLC system (Waters). Peptides were injected onto a 180 µm x 2 cm Symmetry trap (Waters; C18 5 µm 120 Å) and separated on a 100 µm x 10 cm BEH130 column (Waters; 1.7 µm C18 120 Å). After targeted peptide transition optimization and retention time scheduling, 96 transitions were targeted over a 30 min gradient from 1 to 50 % of 99.9% ACN and 1% FA.

The mass spectrometry proteomics data have been deposited to the ProteomeXchange Consortium^25^ via the PRIDE partner repository with the dataset identifier PXD002725 for in-vitro study and PXD007083 for human tissue specimen study.

### Protein identification

Spectral libraries for SWATH-MS quantitation were generated with ProteinPilot^TM^ software 5.0 using the Paragon^TM^ algorithm (SCIEX) in the thorough ID mode including biological modifications and chemical modifications. MS/MS data were searched against the human UniProt database (release February 2016, 20198 entries) with carbamidomethyl as a fixed modification for cysteine residues. An Unused Score cut-off was set to 0.05 and the FDR analysis was enabled.

### Data analysis

Generated Paragon group files were imported into PeakView^TM^ software 2.1 using the SWATH MicroApp 2.0 (release 25/08/2014) to generate a sample specific spectral library which was matched against SWATH-MS data. After retention time calibration with endogenous peptides, data were processed using following processing settings; 100 maximal peptides per protein, maximal 6 transitions per peptide, peptide confidence threshold of 99%, transition false discovery rate <1%, 5 min extraction window and fragment extraction tolerance of 75 ppm. Transition, peptide and protein areas of processed data were exported. Protein areas were log2 transformed and normalized by subtracting median protein areas per sample (Supplementary Fig. S1) and were further analyzed using Perseus software version 2.5 to perform principal component analysis, Student’s *T*-test analysis with permutation based multi-variant testing and hieratical clustering.

SRM data were processed using Skyline software version 2.5. After manual validation of transition peak integration, quantifier peptide peak areas were exported and CVs and peak area rations were calculated.

### Patient clinical data and gene expression data from genomic data commons

We downloaded patient clinical information and gene expression data from Illumina mRNA sequencing of melanoma specimens submitted by the Westmead Hospital, University of Sydney, Australia to the NIH Genomic Data Commons as part of the Skin Cutaneous Melanoma project with the case submitter identifier TCGA-EE- from the TCGA-SKCM project. To compare with the proteomics data, data from AJCC Stage III specimens with either *BRAF* or *NRAS* mutations were used (*n* = 69). Survival time from surgery to last follow up and vital status were extracted. Individual files per patient containing fragments per kilobase of transcript per million mapped reads (FPKM) normalized gene transcripts were combined and transcriptional levels of ICAM-1, PMEL, and ITGAV were extracted. Values were log2 transformed and each gene was normalized by its mean gene transcript level across all patients.

## Results

### Melanoma cell lines

Nine genotyped cutaneous AJCC Stage III early passage melanoma cell lines were selected which broadly represents common clinically relevant mutations in the MAPK pathway (Supplementary Table 1).^24^ Three cell lines possessed an activating *NRAS* mutation (Q61K/L) (C002, C054, and C096), three possessed *BRAF* V600E/K mutations (C078, C088, and C045), and three cell lines were wild type for *BRAF* and *NRAS* (C037, C052, and C084). We also tested the cell line D22M obtained from an AJCC Stage IV patient which had mutations in both *BRAF* V600K and *MEK1* P124L.

### Phenotyping of melanoma cell lines by SWATH mass spectrometry

**Fig. 1:**
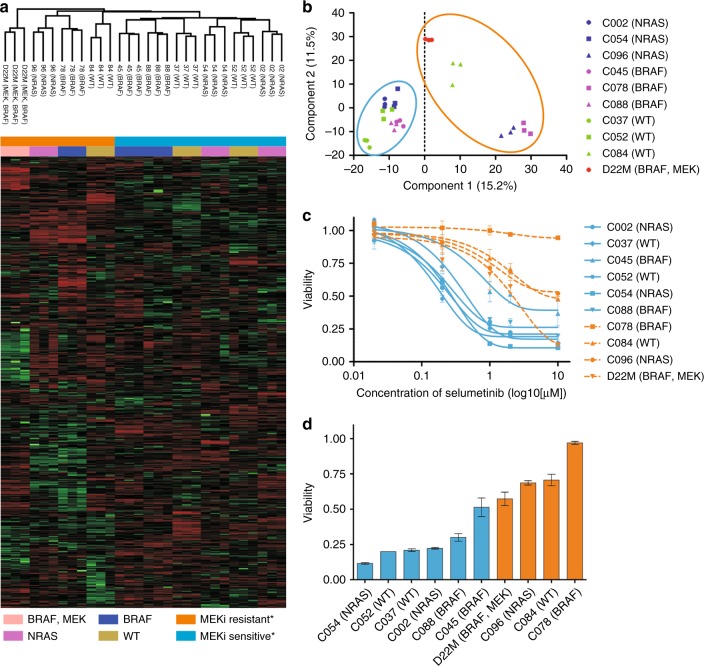
**a** Unsupervised hierarchical clustering of normalized and *z*-score transformed SWATH-MS protein peak areas; * classification based on 10 day MEK inhibition cell viability. **b** 2D PCA projection of melanoma cell lines based on SWATH-MS protein peak areas identifies two clusters. **c** Concentration response curves of the ten melanoma cell lines after ten-day exposure to MEK inhibitor selumetinib (AZD6244). **d** Cell viability after 10 day exposure to 2 µM selumetinib (MEKi). Orange—nominal MEKi resistant, Blue—nominal MEKi sensitive based on PCA clusters

The protein expression phenotypes of these melanoma cell lines were investigated by SWATH data independent quantitative mass spectrometry.^23^ This workflow necessitated the development of a reference peptide spectral ion library which we generated by profiling the melanoma cell lines by on-line 2D-LC-MS/MS combining strong cation exchange and reversed phase chromatography in a liquid chromatography-chip format, as described previously.^26^ The peptide spectral data were searched against the human UniProt database to create a melanoma-derived peptide ion library representing 27,908 peptides from 3209 proteins. Separately, three biological replicates of each of the nine melanoma cell lines were profiled by SWATH-MS using 1 h LC-MS data acquisition time. We extracted peptide fragment ion peak areas (false discovery rate <1%) and summed these to the protein level, enabling relative quantitation of 2265 proteins across all ten melanoma cell lines and replicates. Unsupervised hierarchical clustering (Fig. 1a) and principal component analysis (PCA) (Fig. 1b) based on basal protein expression identified two distinct sample groups. Interestingly, segregation of these groups was not based on *BRAF* or *NRAS* genotype.

### Melanoma cell line sensitivity to MEK inhibition

Since SWATH-MS protein phenotyping did not discriminate the melanoma cell lines based on mutational genotype, we investigated this by screening for response to the MEKi selumetinib (AZD6244) which has been shown to be effective in controlling downstream ERK phosphorylation in BRAF and NRAS mutant tumors (Fig. 1c).^27^ We observed an association with the unsupervised grouping seen in Fig. 1a, b, in that these 6 cell lines were the most sensitive to 2 µM selumetinib after 10 days (average 26 ± 12% viable cells). C078 was the most resistant (97% viability), while C084, C096, and D22M showed strong to partial resistance (Fig. 1d). Therefore, basal protein expression levels from early passage Stage III derived melanoma cells was sufficient to approximate the in vitro response to the ATP-competitive MEK inhibitor, selumetinib and this was unrelated to *BRAF/NRAS* mutational genotype.

### Biological processes associated with with in vitro response to MEK inhibition

**Fig. 2:**
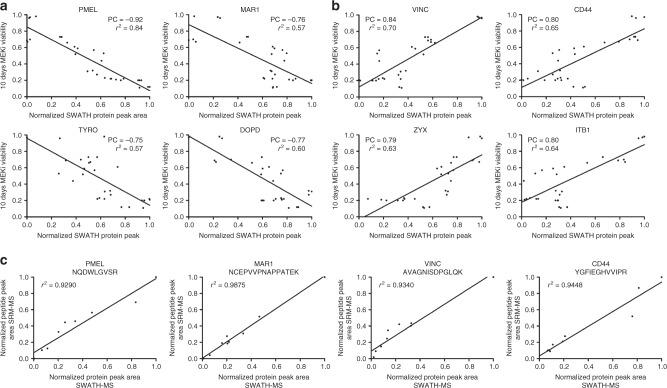
Representative protein expression correlation with cell viability after ten-day exposure to 2 µM selumetinib, **a** positive correlations associated with MEK resistance **b** negative correlations associated with MEK sensitivity. PC, Pearson correlation, *r*^2^, linear regression. **c** Independent measurement of protein abundance by SRM-MS correlates with SWATH-MS measured protein abundance protein peak area (three biological replicates per cell line)

To identify changes in biological processes that account for differential sensitivity to MEK inhibition in these cells, we correlated normalized SWATH protein areas with MEKi cell viability. The average Pearson correlation (PC) for all SWATH protein areas was PC = −0.02 with a standard deviation *σ* = 0.37, thus proteins with PC > 2σ were considered as highly relevant. Using these criteria, 38 proteins positively correlated with MEKi resistance (PC ≥ 0.75), while 27 proteins were negatively correlation with MEKi resistance (PC ≤ −0.75) (Fig. 2 and Supplementary Fig. S2 and Supplementary Table S3). We considered these 63 proteins as key participants in defining metastatic melanoma growth and survival in the presence of selumetinib.

To confirm the SWATH-MS results, 33 peptides from 18 proteins were selected for independent analysis using a targeted MS method, SRM-MS^28^ (Supplementary Table S4). We selected these proteins to represent those which highly correlated with MEKi response in the cell lines and spanned to proposed biological functions described below. This analysis confirmed the reliability of the global SWATH-MS dataset (Supplementary Table S4 and Supplementary Fig. S3).

Protein expression levels which positively correlated with MEKi resistance were those mainly involved in cell motility (e.g., caldesmon and tropomyosin alpha-4 chain), cell adhesion and cell–cell/matrix communication (e.g., integrin alpha-V (ITGAV), integrin beta 1 (ITB1), CD44) and cytoskeletal organization (e.g., fascin 1 (FSCN1), Ras-related protein R-Ras (RRAS) and vinculin (VINC)). Reduced expression levels of proteins associated with MEKi resistance (negative correlations) were observed for those involved in fatty acid biosynthesis including fatty acid synthase (FAS) and the mitochondrial medium-chain specific acyl-CoA dehydrogenase (ACADM). Similar negative correlations were found for some proteins responsible for melanin biosynthesis and melanosome maturation such as melanocyte protein PMEL, melanoma antigen recognized by T-cells 1 (MAR1), microphthalmia-associated transcription factor (MITF), tyrosinase (TYRO) and D-dopachrome decarboxylase (DOPD). The change in levels of the molecular drivers for cell pigmentation was consistent with the appearance of cell lysates.

### MEKi response phenotype in melanoma tissue

**Fig. 3:**
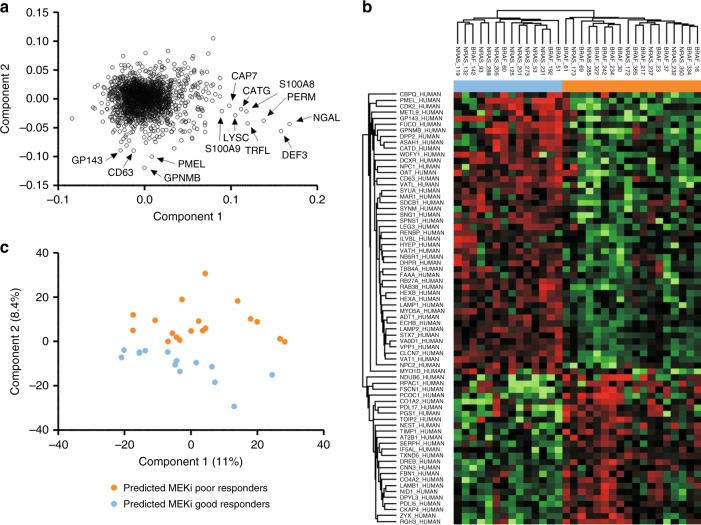
**a** Individual protein loading in principal component 1 and 2 of 1877 quantified proteins across the 32 melanoma tissues with indication of proteins with highest positive loading in component 1 and highest negative loading in component 2; NGAL, neutrophil gelatinase-associated lipocalin; DEF3, neutrophil defensin 3; PERM, myeloperoxidase; CATG, cathepsin G; LYSC, lysozyme C; TRFL, lactotransferrin; CAP7, azurocidin; GPNMB, transmembrane glycoprotein NMB; GP143, G-protein coupled receptor 143. **b** Hierarchical clustering of melanoma tissue samples based on significantly changing proteins from PCA component 2 (*q* value < 0.1 and fold change >2-fold). **c** 2D projection of PCA from SWATH analysis of melanoma tissues with indication of predicted MEKi sensitive (light blue) and MEKi resistant (orange) phenotype

To establish whether the proteins associated with the MEKi response phenotype could be detected directly in patient tumors, we examined 32 specimens (16 *BRAF*, 15 *NRAS*, 1 *BRAF/NRAS* wild type; Supplementary Table 2) where survival time was defined as “good” or “poor”. The specimens annotated as “good” were from patients with >4 years survival (*n* = 18, mean post biopsy survival 107.5 months with 14 patients alive on last follow-up), and those as “poor”, from patients with mean melanoma specific survival of <6.7 months (*n* = 14). We used the SWATH library established from melanoma cell lines to measure protein expression in these tissue specimens, allowing 1877 proteins to be quantified among all 32 tissue samples (Supplementary Table S5 and Supplementary Fig. S4). PCA analysis based on the expression levels of all 1877 proteins did not cluster specimens by *BRAF/NRAS* genotype, nor “good/poor” survival. Greatest variance which was observed by the first principal component stratified tumors due to the abundance of invading neutrophils, with neutrophil gelatinase-associated lipocalin, neutrophil defensin 3, myeloperoxidase, anti-microbial proteins cathepsin G, Lysozyme C, lactotransferrin and azurocidin, and S100 proteins A8 and A9 showing the highest positive loading (Fig. 3a). The second principle component however separated the tumors according to proteins associated with melanocyte differentiation (highest negative loading for PMEL, transmembrane glycoprotein NMB, G-protein coupled receptor 143 and CD63) (Fig. 3a). Hence, FDR corrected two sample *T*-test analysis (*q*-value < 0.1 and fold change >2-fold) (Fig. 3b) was performed comparing tumors with negative principal component 2 (PC2) projections to tumors with positive PC2 projections (Fig. 3c). Proteins identified in higher abundance in the tumors with negative PC2 were involved in melanogenesis, for example, PMEL (which negatively correlated with the MEKi resistance phenotype in cell lines) and proteins with lower abundance were those which positively correlated with the MEKi resistance phenotype observed in melanoma cell lines (i.e., Fascin 1, PDLIM5, PDLIM7, and Zyxin) (see Fig. 2 and Supplementary Figure S3). Therefore, we conclude that protein expression phenotypes established from fresh-frozen melanoma specimens resemble those observed in melanoma cells lines which correlated with in vitro selumetinib response.

### Abundance of ICAM-1 and ITGAV are associated with patient survival

**Fig. 4:**
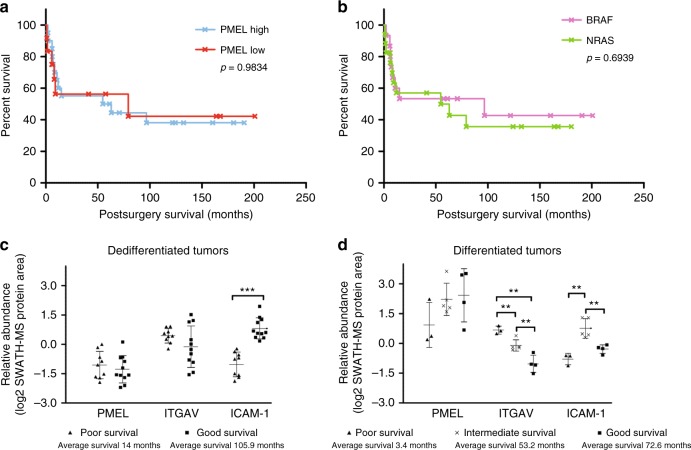
Kaplan–Meier patient survival plot (*n* = 32), shown from time of surgery of specimen collection based on **a** high/low PMEL protein expression and **b** oncogenic *NRAS/BRAF* genotype; log rank test. Relative protein abundance of PMEL, ICAM-1, and ITGAV associated with survival time in **c** de-differentiated (low PMEL expression) and **d** differentiated (high PMEL expression) tumors; 2 sample *T*-test, mean protein area with standard deviation

**Fig. 5:**
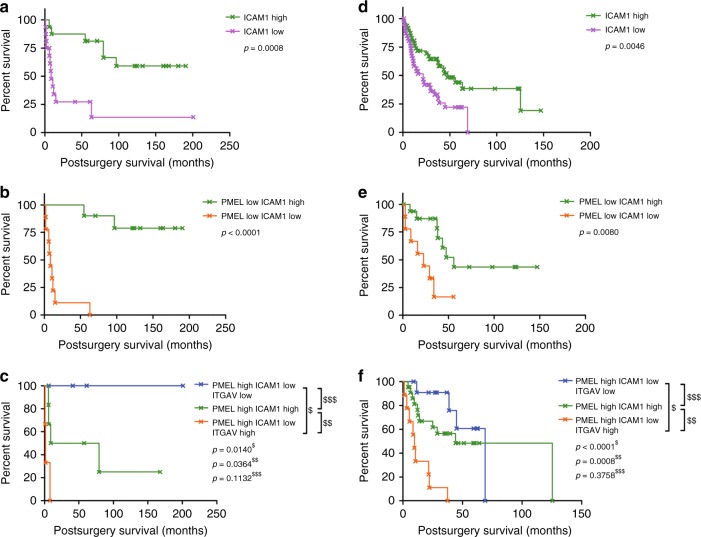
Kaplan–Meier patient survival plot (*n* = 32) based on SWATH-MS protein area of (**a**) ICAM-1 alone, **b** ICAM-1 in dedifferentiated tumor specimen (low PMEL expression) and **c** ICAM-1 and ITGAV in differentiated tumor specimen (high PMEL expression). Kaplan–Meier patient survival plot of 69 AJCC Stage III tumor specimens in the GDC database (TCGA-SKCM; EE annotated cases) based on FPKM normalized transcription level of (**d**) ICAM-1 alone, **e** ICAM-1 in dedifferentiated tumor specimens (low PMEL expression), and **f** ICAM-1 and ITGAV in differentiated tumor specimens (high PMEL expression)

As high PMEL appears to be a feature of sensitivity to MEKi it was of interest to examine the prognostic utility of this protein in melanoma tissue specimens. We observed a range of PMEL abundance levels in various lymph node tumor tissues (mean 7-fold difference, Supplementary Table S6), however, PMEL expression alone was not associated with differences in post-surgery MSS (*p* = 0.98, Fig. 4a), nor was *BRAF/NRAS* genotype (*p* = 0.69, Fig. 4b). To search for other proteins with greater prognostic utility, two-sample *T*-tests comparing “good” versus “poor” post-surgery survivors was performed (Supplementary Table 5). Patients with poor survival showed reduced expression of intercellular adhesion molecule 1 (ICAM-1, also known as CD54, *p* = 3.4e−4, 2.2-fold, Fig. 4c, d) and Kaplan–Meier survival analysis revealed significant MSS advantage (*p* = 8e−4, HR = 6.0, 95% CI 2.1–17.6, Table 1) for patients with high ICAM-1 protein expression (Fig. 5a). Interestingly, patients with low ICAM-1 levels had a higher metastatic burden, with 81% of these patients developing distant metastases compared to 37.5% for patients with high ICAM-1 levels. We further noted that the expression of ICAM-1 in de-differentiated tumors, defined as those with low PMEL expression (Supplementary Table 5), was a highly significant prognostic factor, where low ICAM-1 expression correlated with diminished MSS (*p* < 0.0001, HR 25.1, 95% CI 6.0–104.6) with median survival of 8.4 months (*n* = 10) for low ICAM-1 expression compared with an estimated 128.4 months (*n* = 9) for high ICAM-1 expression (Fig. 5b). In contrast, in pigmented melanoma (i.e., differentiated with high PMEL expression) (Supplementary Table S5), ICAM-1 alone was not statistically different between good or poor post-surgery survivors (Student’s *T*-test *p* = 0.56), however, low ICAM-1 expression and high expression of the vitronectin receptor integrin alpha-V (ITGAV, CD51) were prognostic for very short median MSS of 1.4 months (Fig. 5c). We note that ITGAV was in our panel of response markers of MEKi resistance. Interestingly, this phenotype coincided with lower expression of beta catenin (4.9-fold), RAB27A (2.2-fold) and the methyltransferase-like protein 9 (METL9, 15-fold). There were four tumors with low expression of ITGAV in low ICAM-1 pigmented specimens and these patients showed a median survival of 51.1 months. In the pigmented tumors where high ICAM-1 is maintained, MSS was independent of ITGAV (Student’s *T*-test *p* = 0.33) and a median survival of 33.2 months (*n* = 6) was observed.

**Table 1:** Post-surgery survival analysis of AJCC Stage III/IV melanoma specimens investigated by SWATH-MS and downloaded from publicly available transcript data sets grouped based on presence and absence of ICAM-1, PMEL, and ITGAV

Expression of marker	SWATH-MS proteins				TCGA-SKCM; EE cases—mRNA transcripts			
	*p*-value	Hazard ratio	95% CI	Median survival (months)	*p*-value	Hazard ratio	95% CI	Median survival (months)
ICAM-1 low vs. ICAM-1 high	0.0008	6.0	2.1–17.6	8.2 vs. 109.1	0.0046	2.2	1.3–3.8	21.6 vs. 47.5
ICAM-1 low, PMEL low vs. ICAM-1 high, PMEL low	<0.0001	25.1	6.0–104.6	8.4 vs. 128.4	0.008	5.9	1.6–22.2	22.9 vs. 55.9
ICAM-1 low, PMEL high, ITGAV high vs. ICAM-1 low, PMEL high, ITGAV low	0.014	20.8	1.8–235.0	1.4 vs. 51.1	<0.0001	22.5	5.5–90.8	9.7 vs. 69.1
ICAM-1 low, PMEL high, ITGAV high vs. ICAM-1 high, PMEL high	0.036	9.9	1.2–85.2	1.4 vs. 33.2	0.0008	7.6	2.3–25.1	9.7 vs. 44.2
ICAM-1 low, PMEL high, ITGAV low vs. ICAM-1 high, PMEL high	0.113	0.2	0.02–1.5	51.1 vs. 33.2	0.376	0.6	0.2 – 1.8	69.1 vs. 44.2

We further assessed the prognostic value of the three markers for relapse free survival (RFS) (Supplementary Table S7) which demonstrated favorable outcomes RFS for high ICAM-1 protein levels in the 26 AJCC Stage III patients who were disease free after surgery (*p* < 0.0001, Supplementary Fig. S5a). This RFS for ICAM-1 was also observed when we grouped patients based on PMEL status, with higher ICAM-1 expression being advantageous (*p* < 0.0001, Supplementary Fig. S5b). In PMEL low tumors, disease free survival was not associated with ICAM-1 and ITGAV levels, (Supplementary Fig. S5c).

### Survival based on RNA transcript level is consistent with SWATH-MS based prognosis in *NRAS/BRAF* mutant melanoma

We sort to validate the proteomic prognostic biomarkers by examining ICAM-1, PMEL, and ITGAV mRNA transcript levels from 69 AJCC Stage III melanoma tissue specimens with either BRAF V600E/G/K/R or NRAS Q61H/K/R mutations that were submitted to TCGA and subsequently extracted from the NIH Genomic Data Commons database using the TCGA-SKCM EE cases identifier (https://portal.gdc.cancer.gov/projects/TCGA-SKCM). (Fig. 5d–f, and Supplementary Table S8). Kaplan-Meier survival analysis based on gene transcript levels showed similar trends as observed for SWATH-MS protein level measurements (Fig. 5a–c), confirming that higher ICAM-1 transcript or protein showed a survival advantage (Table 1). Further, analysis of mRNA transcript levels for all 202 BRAF or NRAS mutant AJCC Stage III specimens in the larger TCGA-SKCM dataset confirmed this finding (Supplementary Fig. S6).

Further subgrouping of the TCGA-EE dataset based on pigmentation status assessed from PMEL transcript level also confirmed longer survival of patients with low pigmented specimens and high ICAM-1 levels (*p* = 0.008, *n* = 26; Fig. 5e). In pigmented specimens, the prognostic associations measured by SWATH-MS were consistent with those based on gene transcript levels, with significant survival benefit associated with low ITGAV in pigmented, ICAM-1 low tumors (*p* < 0.0001, HR = 22.5, *n* = 21; Fig. 5f). In high ICAM-1 compared with low ICAM-1 pigmented tumors, the ITGAV transcript level was not prognostic.

Finally, we observed that in the 18 cases with wildtype *NRAS/BRAF* from the TCGA-EE dataset, ICAM-1 mRNA transcript level was not a prognostic factor (Supplementary Fig S7).

## Discussion

In this study, we demonstrated that mass spectrometry-based protein profiling of early passage cell lines derived from Stage III cutaneous melanoma patients growing under basal conditions displayed a protein expression pattern that correlated with in vitro response to selumitinib, but was not correlated with *NRAS/BRAF* genotype. This observation is consistent with the clinical experience that not all BRAF-mutant melanomas are sensitive to BRAF inhibition or MEKi^11^ and suggests that response to MAPK pathway blockade is reflected in the expression of particular proteins. In contrast, the *NRAS/BRAF* genotype was not a predictive factor in determining in vitro cell response to MEKi, nor patient prognosis. Gene ontology based descriptions of the biological functions linked to these proteins finds involvement in cell motility, adhesion, cytoskeletal architecture, fatty acid metabolism and melanosome maturation.

It is well established that there are several mechanisms in melanoma that result in a poor initial and/or durable response to kinase inhibitor therapy.^29^ So far, the majority of these studies focus on MAPK and other complementary signaling pathways able to maintain or switch between a proliferative or invasive state in response to targeted inhibition of key effectors.^15,16,30,31^ Here, we demonstrated that the growth response to MEKi in early cultures of Stage III melanoma cells depends on intrinsic global protein expression programs that provide distinct cellular states that are independent of *NRAS/BRAF* mutation or prior exposure to MEKi. Intrinsic resistance to inhibitors has been observed previously^18,32,33^ and linked to tumor stromal plasticity or transcriptional programs that reflect metastatic potential, or dependence on MAPK pathway activation. We observed a proteomic profile for response to MEKi that positively correlated with the expression of microphthalmia-associated transcription factor (MITF) which is often found to be suppressed in BRAF V600 mutated cells.^34^ Depending on the expression level, MITF can act in a pro-growth (low) or anti-proliferative (high) manner. We observed MITF expression to be low in MEKi resistant cells and confirmed that its translational activity is impinged as evidenced by the low expression of several melanosomal proteins that are targets of MITF transcriptional activity (Supplementary Table S9).

Alongside the expression of the MITF transcriptional network, the unbiased measure of the proteome provided by SWATH-MS identified other proteins correlated with intrinsic resistance to MEKi in cell lines. We observed positive correlations with intrinsic MEKi resistance within a network of ECM and cytoskeletal proteins involved in motility and cell adhesion, several of which have been previously associated with invasiveness in melanoma.^20^ One of these proteins the hyaluronic acid receptor, CD44, is a cell surface transmembrane protein found elevated in melanoma metastases.^35,36^ CD44 receptor stimulation by ECM components contributes to matrix adhesion, migration, growth promotion, and cell survival in several cancers including melanoma.^37–39^ Isoforms of CD44 are known to orchestrate multiple phenotypes in melanoma altering in expression through cleavage and splicing in response to extracellular or environmental signaling.^40–42^ ECM signaling through CD44 involves the receptor complexes ERBB4/HER and HGF/C-met that signal through RAS, Rho/rac, MAPK, and AKT pathways to promote tumorigenicity.^43,44^ In melanoma cells, metabolic plasticity can provide cellular states that aid tumor progression and provide survival mechanisms in response to microenvironmental changes and drug therapy.^45^ For example, intrinsic sub-populations of slow cycling metabolically distinct cells that facilitate multi-drug resistance (e.g., proteasome inhibition or MAPK pathway inhibition) exhibit increased oxidative phosphorylation (OXPHOS).^46^ Our data suggests that reduced biosynthesis of fatty acids associated with low expression of FAS and ACADM is an intrinsic property of MEKi resistant melanoma cells. FAS is well-known as an oncogene expressed in rapidly dividing tumors.^47^ Whilst the majority of variation in the proteome measured by SWATH-MS reflected an expression gradient that correlated with MEKi response, further sub-stratification of tumor cells was also possible. For example, the MEKi resistant cell lines C078 and C096 exhibited high expression of proteins Cav-1, PRTF, and BASP1 indicating a cellular phenotype with higher amounts of lipid-raft membrane micro-domains. Lipid rafts containing Cav-1 and the intergrins ITGAV and ITB1 (also found elevated in MEKi resistant cells) can lead to signaling through the PI3K/AKT axis^48^ and provide alternative pro-growth signals independent of MAPK pathway.

The identification of MEKi sensitive phenotypes from Stage III tumors (33% of good survivors and 43% in poor survivors), which among others include changes to melanogenesis (higher PMEL expression and other melanosome specific proteins) suggests this phenotype may have utility for rationale selection of MEKi sensitive patients. However, when we tested the utility of the combined in vitro MEKi response panel of all 63 proteins to predict post-surgery survival in untreated patients the association failed. Nonetheless, univariate analysis clearly illustrated the prognostic value of ICAM-1 for MSS in *NRAS/BRAF* melanoma (Fig. 5), where reduced expression is associated with worst survival, a finding that has not been widely reported in melanoma. We further revealed the prognostic utility of combining ICAM-1 expression with the differentiation marker PMEL and the cell adhesion protein ITGAV for more specific prognostication of molecular subtypes.

Our analysis focused on melanoma cases driven by mutation of *NRAS/BRAF* genes as these are the most prevalent driver mutations in melanoma.^1,2^ When we examined the smaller number of wildtype *NRAS/BRAF* cases in the TCGA-SKCM dataset it was interesting to note that ICAM-1 mRNA transcript levels were not prognostic (Supplementary Fig. S7), directly contrasting the situation in *NRAS/BRAF* mutated melanoma where this marker is a strong prognosticator. We speculate that the differentiation status of wildtype *NRAS/BRAF* melanomas impacts on the prognostic utility of ICAM-1, as more than three quarters of the wildtype *NRAS/BRAF* tumors had high PMEL levels and thus were derived from well differentiated melanocytes. As we discovered in the *NRAS/BRAF* mutant tumors derived from well differentiated melanocytes (high PMEL), ICAM-1 expression alone was not a biomarker, with prognosis requiring consideration of ITGAV expression in these tumors (Fig. 5f). Insufficient cases were available to assess ITGAV transcripts as a biomarker in wildtype *NRAS/BRAF* melanoma.

There remains confounding information on the relationship of ICAM-1 expression and prognosis in a range of cancers. In a study on non-Hodgkin’s lymphoma, improved survival was observed for patients with relatively high ICAM-1 expression,^49^ as was the case in breast cancer where ICAM-1 was associated with low growth potential and negative lymph nodes involvement.^50^ In contrast, high levels of the soluble ICAM-1 (sICAM-1), which acts as an antagonist of ICAM-1 functions, has been identified in patients with higher tumor burden and faster progressing tumors in colorectal, gastric cancer and diffused large B-cell lymphoma.^51–53^ It has been well established that ICAM-1 binding to lymphocyte function-associated antigen 1 (LFA1) regulates cytotoxic T lymphocytes (CTLs) cytotoxicity.^54^ In support of our findings, Anastassiou and colleagues^55^ reported that the loss of ICAM-1 in uveal melanoma was associated with increased risk of metastasis 5 years beyond diagnosis. Hamai and colleagues^56^ demonstrated that reduced susceptibility of metastatic melanoma to CTL lysis is linked to down-regulation of ICAM-1 expression. One interpretation of the improved prognosis seen in our study of patients with higher ICAM-1 is that these tumors interface with immune surveillance, contributing in part to controlling metastasis and hence prolonging survival.

Poor survival in patients with poorly differentiated (low PMEL expression) tumors was linked with low ICAM-1 expression (median post-surgery survival 8.4 months, HR = 25.1, *p* < 0.0001), and this was confirmed based on the mRNA transcript level in a separate dataset (Fig. 5; Table 1). It was noteworthy that these specimens also showed relatively high levels of ITGAV (Supplementary Fig. S8). ITGAV was also highly expressed in the group with poorest survival in pigmented tumors (see below). The loss of ICAM-1 in melanoma cells, which has been used to distinguish between primary and metastatic melanoma, promotes PI3K/AKT signaling and thereby protection from lysis by melanoma antigen probed CTLs, however, treatment with interferon gamma can induce ICAM-1 expression on these metastatic melanomas and reestablish susceptibility to CTL lysis.^56^ It is tantalizing to suggest that interferon gamma treatment to prime CTLs, coupled with immunotherapy may be a useful approach to treat melanoma patients with low ICAM-1 and low PMEL expression. This is because patients with this phenotype have been shown here to have very short survival and since low PMEL expression is one of the driving factors of the ‘MEKi resistant’ phenotype observed in vitro, these patients would most likely not have benefited from MEK kinase inhibition treatment alone.

In pigmented metastatic tumors, high expression of ITGAV in specimens possessing low ICAM-1 was associated with dismal survival (median post-surgery survival 1.4 months, HR = 20.0, *p* = 0.014). Similar survival trends were also observed for this molecular phenotype using the TCGA-SKCM dataset mRNA transcript level information of Stage III *NRAS/BRAF* melanoma specimens (Fig. 5; Table 1). High ITGAV expression along with high integrin alpha-3 and alpha-6 subunits measured by immunohistochemistry, has previously been associated with reduced survival in colorectal cancer.^57^ The ICAM-1 low, ITGAV high phenotype seen here in pigmented melanoma that is associated with dismal survival coincided with the loss of RAB27A, a protein involved in melanosome transport and excretion. RAB27A expression correlates with melanosome maturation, with lowest levels in stage I and highest in stage IV melanosomes.^58^ Low levels of RAB27A seen in tumors with high PMEL expression suggests malfunctioning melanosome maturation and stagnation in the early stages I–III. Interestingly, high levels of stage I–III melanosomes have been linked to cisplatin resistance in highly pigmented MNT-1 melanoma cells due to drug trapping inside the melanosomes.^59^ In our study of metastatic melanoma specimens, disrupted melanin production was associated with poor patient survival; if validated in earlier stage disease in a larger patient cohort, these markers of stagnating melanosome maturation (high ITGAV expression in high PMEL-expressing tumors and the loss of RAB27A) might indicate a causative role for this phenotype of poor survival. Since these tumors still have protein hallmarks of the MEKi sensitive phenotype observed in the cell line cohort, these patients might have benefitted from treatment with MEKi drug therapy if it had been available at the time of their diagnosis. Importantly, patient selection based on PMEL/ITGAV expression could be particularly relevant given promising preliminary approaches of combining MAPKi with anti-PD-L1/PD-1 immunotherapy.^60,61^

This study has demonstrated the value of protein phenotyping of in vitro cell line models and patient tissue from lymph node melanoma metastases to inform likelihood of response to MEKi. In cell lines treated with selumitinib *NRAS/BRAF* genotype was not useful in predicting drug response. We then assessed the use of specific protein expression biomarkers in *NRAS/BRAF* metastatic tissue specimens for prognostication, which identified a three biomarker panel that showed clear associations with survival at both mRNA transcript and protein level (Fig. 5). The SWATH-MS screening approach that we carried-out is arguably more rapid than IHC detection used in pathology laboratories. Taking approximately 1 h to acquire MS data per sample, SWATH-MS has the utility to catalog every patient sample/cell line collected in the clinic^62^ and when combined with measures of disease phenotype may identify specific profiles or correlates to aid clinical decision making in support of personalized treatments. Currently, tissue biopsy and phenotypic classification of melanoma is based upon morphology (Breslow tumor thickness, tumor ulceration) and metastatic involvement of lymph nodes. This information is used to indicate prognostic outcome and informs clinical treatment regimens. More recently, targeted gene mutation analysis or genome sequencing has been useful in selecting patients for specific kinase inhibitor therapy. Whilst genotyping has its place, it does not fully capture the complexity and heterogeneity of the disease process and is ultimately limited when treating a disease of high plasticity as seen in melanoma. Our study and others^12,14,18,33,46,63–65^ clearly demonstrate the power of protein/gene expression profiling in revealing the molecular links underpinning different melanoma phenotypes and expanding the use of these tools into large cohorts holds potential to unlock more therapeutic regimens based on molecular landscapes.

### Data availability

The mass spectrometry proteomics data have been deposited to the ProteomeXchange Consortium^25^ via the PRIDE partner repository with the dataset identifier PXD002725 for in-vitro study and PXD007083 for human tissue specimen study.

## Electronic supplementary material

Supplementary Figures and Tables

Supplementary Tables S3-S5
