# Supplementary material for: Proteomic phenotyping of metastatic melanoma reveals putative signatures of MEK inhibitor response and prognosis
Source: Br J Cancer. 2018 Aug 17;119(6):713–23. doi: 10.1038/s41416-018-0227-2 (PMC6173697; doi:10.1038/s41416-018-0227-2)
Supplement: Supplementary file 1 — Supplementary Figures and Tables [file 41416_2018_227_MOESM1_ESM.docx]

**Supplementary Material**

**Proteomic phenotyping of metastatic melanoma reveals putative signatures of MEK inhibitor response and prognosis**

Christoph Krisp^1^, Robert Parker^1^, Dana Pascovici^1^, Nicholas K. Hayward^2^, James S. Wilmott^3^, John F. Thompson^3,4^, Graham J. Mann^3^, Georgina V. Long^3,5^, Richard A. Scolyer^3,4^, Mark P. Molloy^1,6†^

**Supplementary Figures (Supp F1-F8)**

**Supplementary Tables (Supp T1-T2, T6-T9)**

**Supplementary Tables in Excel format (Supp T3-T5)**

**Supplementary Figure S1:** **(a)** Box plot of log2 transformed protein peak area of all quantifiable proteins in cell lines established from AJCC Stage III/IV cutaneous melanomas, **(b)** box plot of log2 – median log2 (all proteins per sample) normalized protein peak area. **(c)** SWATH-MS profiles from 1h RP-LC-MS analysis of ten melanoma cell lines analyzed in 3 technical replicates.

**Supplementary Figure S2:** 10-day MEKi cell viability versus SWATH-MS proteins area correlation (0 to 1 normalized) of all proteins with correlation > 2 times the standard deviation of all proteins

Continuation of Supplementary Figure S2

Continuation of Supplementary Figure S2

Continuation of Supplementary Figure S2

**Supplementary Figure S3:** SRM-MS peptide area versus SWATH-MS protein area correlation (0 to 1 normalized) of all peptides selected for SRM validation.

Continuation of Supplementary Figure S3

**Supplementary Figure S4:** Box plot of log2 transformed protein peak area of all quantified proteins in the 32 fresh frozen AJCC Stage III metastatic melanoma specimens, **(b)** box plot of log2 – median log2 (all proteins per sample) normalized protein peak area.

**Supplementary Figure S5:** Kaplan – Meier patient relapse free survival of 26 AJCC Stage III tumor specimens (disease free patients after resection surgery) based on SWATH-MS protein area of (**a**) ICAM-1 alone, (**b**) ICAM-1 in dedifferentiated tumor specimen (low PMEL expression) and (**c**) ICAM-1 and ITGAV in differentiated tumor specimen (high PMEL expression).

**Supplementary Figure S6:** Kaplan – Meier post-surgery survival analysis based on ICAM-1 mRNA levels in 202 AJCC Stage III melanoma with BRAF or NRAS mutation as part of the TCGA-SKCM data set submitted to the GDC data base. Patients with high ICAM-1 expression had median post-surgery survival of 148 months (> 12 years) whereas patients with ICAM-1 low expression had a median survival of 61 months (5 years).

**Supplementary Figure S7:** Kaplan – Meier post-surgery survival analysis based on ICAM-1 mRNA levels in 18 AJCC Stage III melanoma with BRAF and NRAS wild type as part of the TCGA-SKCM-EE annotated TCGA-SKCM data set submitted to the GDC data base. No association with survival could be demonstrated based on ICAM-1 transcript.

**Supplementary Figure S8**: Relative abundance of ITGAV in dedifferentiated (low PMEL expression level) AJCC Stage III melanoma in ICAM-1 high (median post-surgery survival > 4 years) and ICAM-1 low (median post-surgery survival < 4 years) in specimens profiled by SWATH-MS and in mRNA transcript levels in the TCGA-EE- data set.

**Supplementary Table 1:** Patient characteristics of derived C0 series cell lines. Gene mutations reported previously [^24^](#_ENREF_24)^,^ [^65^](#_ENREF_65). * Homozygous gene deletion, ^#^ Amplification, n/a = unknown, n/d = non detected, ^cell line established from metastasis, ^§^ survival from vaccination

| Cell Line | Gender | Site of primary | Site of specimen | Age (onset) | AJCC Stage | Death after surgery (weeks) | Alive after surgery (weeks) | Mutated gene(s) | Mutation detected |
| --- | --- | --- | --- | --- | --- | --- | --- | --- | --- |
| C002 | M | R upper leg | Lymph node  R thigh | 59 | III | 76 | n/a | *NRAS*  *P16NK4A*  *P14ARF* | Q61K  HD*  HD* |
| C054 | F | R lower leg | Lymph node  R groin | 51 | III | n/a | 535 | *NRAS*  *P16NK4A* | Q61K  HD* |
| C096 | M | R lower leg | Lymph node  groin | 44 | III | 157 | n/a | *NRAS*  *P16NK4A*  *MAP3K9* | Q61R  HD*  S533Y |
| C078 | M | L lower back | Lymph node  axillary | 56 | III | 68 | n/a | *BRAF*  *BMPR1A*  *MITF* | V600E  Y245S  AMP-L^#^ |
| C045 | F | R shoulder | Lymph node  R axilla | 21 | III | 180 | n/a | *BRAF*  *TP53* | V600E  A138V |
| C088 | F | R upper abdomen | Lymph node  R axilla | 55 | III | n/a | 457 | *BRAF* | V600K |
| C037 | F | n/a | Lymph node  R axilla | 27 | III | n/a | 569 | n/d | — |
| C052 | M | mid back | Lymph node  L axilla | 69 | III | n/a | 537 | *MITF*  *CCND1* | AMP-L^#^  HD* |
| C084 | M | R upper back | Lymph node  R neck | 75 | III | 88 | n/a | *MAP3K9* | G600R  R827Q  9963S |
| D22M | M | R shoulder | Subcutaneous metastasis to the sternum^ | 46 | IV | 41^§^ | n/a | *BRAF*  *MEK1* | V600K  P124L |

**Supplementary Table 2:** Tumor pathology and patient survival; * indicates last known status as alive, # indicates treatment prior to specimen collection. ^ Based on correlation of protein expression with MEKi response phenotype observed with the melanoma cell lines; CVac = Cancer vaccine; DNCB = 2,4-dinitrochlorobenzene; DTIC = dimethyltriazeno-imidazol carboxamide; VMCL = vaccinia melanoma cell lysate; WBRT = whole brain radiotherapy

| Patient | Site of primary | Gender | Age (primary diagnosis) | AJCC Stage (surgical resection) | Specimen Type | Time to death after surgery (months) | Alive after surgery (months) | Gene mutated | Mutation | Predicted response to MEK inhibition^ | Treatment strategy after surgery (if not indicated otherwise) |
| --- | --- | --- | --- | --- | --- | --- | --- | --- | --- | --- | --- |
| *Patients with good survival* | | | | | | | | | | |  |
| 1 | Occult | F | 44 | III | Regional lymph nodes |  | 171.14 | BRAF | L397L, L397L | **good** | VMCL vaccine |
| 2 | Leg | F | 68 | IIIB/IIIC | Regional lymph nodes |  | 134.60 | NRAS | Q61K | poor | - |
| 3 | Occult | M | 40 | III | Regional lymph nodes |  | 132.27 | NRAS | Q61R | poor | VMCL vaccine |
| 4 | Leg | F | 46 | IV | Distant lymph nodes |  | 127.84 | NRAS | Q61K | poor | CVac |
| 5 | Leg | F | 19 | IIIC | Regional lymph nodes |  | 124.16 | BRAF | V600E | poor | adjuvant radiotherapy |
| 6 | Occult | M | 30 | III | Regional lymph nodes |  | 121.69 | BRAF | V600E | poor | radiotherapy R axilla |
| 7 | Trunk | M | 71 | IIIB | Regional lymph nodes |  | 120.77 | NRAS | Q61K | poor | autologous DNCB vaccine |
| 8 | Trunk | M | 32 | IIIC | Regional lymph nodes |  | 114.50 | NRAS | Q61K | **good** | adjuvant radiotherapy |
| 9 | Trunk | M | 62 | IIIC | Regional lymph nodes |  | 97.54* | BRAF | V600E | poor | CVac |
| 10 | Leg | M | 47 | IIIC | Regional lymph nodes | 96.56 |  | BRAF | V600E | poor | adjuvant radiotherapy |
| 11 | Arm | M | 28 | IV | Regional lymph nodes | 79.11 |  | NRAS | Q61R | poor | VMCL vaccine #; adjuvant radiotherapy |
| 12 | Trunk | F | 48 | IIIC | Regional lymph nodes |  | 70.67* | BRAF | V600E | poor | - |
| 13 | Trunk | M | 63 | IIIB | Regional lymph nodes | 62.85 |  | NRAS | Q61R | **good** | adjuvant radiotherapy |
| 14 | Trunk | M | 56 | IV | Distant lymph nodes |  | 61.17* | BRAF | V600K | **good** | VMCL vaccine#; palliative radiotherapy |
| 15 | Arm | M | 50 | IIIB | Regional lymph nodes |  | 57.43 | BRAF | V600R | poor | - |
| 16 | Trunk | F | 63 | IIIC | Regional lymph nodes | 54.60 |  | NRAS | Q61H | poor | adjuvant radiotherapy |
| 17 | Occult | M | 24 | III | Regional lymph nodes |  | 41.10* | NRAS | Q61K | **good** | - |
| 18 | Trunk | F | 56 | IIIB | Field Skin & Soft Tissue |  | 6.01* | NRAS | Q61K | **good** | adjuvant radiotherapy |
| *Patients with poor survival* | | | | | | | | | |  |  |
| 1 | Arm | F | 56 | IIIC | Regional lymph nodes | 14.95 |  | BRAF | V600E | poor | CVac and WBRT |
| 2 | Leg | F | 56 | IIIB | Regional lymph nodes | 11.89 |  | NRAS | Q61R | poor | Chemotherapy for Breast Carcinoma # |
| 3 | Leg | F | 48 | IIIB/IIIC | Regional lymph nodes | 10.71 |  | BRAF | V600E | poor | palliative radiotherapy |
| 4 | Leg | M | 53 | IIIC | Regional lymph nodes | 9.03 |  | NRAS | Q61R | **good** | adjuvant radiotherapy / DITC |
| 5 | Trunk | M | 49 | IIIC | Regional lymph nodes | 8.41 |  | BRAF | V600E | poor | adjuvant radiotherapy |
| 6 | Trunk | M | 64 | IIIC | Regional lymph nodes | 7.92 |  | NRAS | Q61R | **good** | adjuvant radiotherapy |
| 7 | Trunk | M | 66 | IIIC | Regional lymph nodes | 7.06 |  | BRAF | V600E | **good** | CVac and WBRT |
| 8 | Leg | M | 53 | IIIC | Regional lymph nodes | 6.21 |  | BRAF | V600E | poor | CVac and radiotherapy |
| 9 | Trunk | M | 47 | IV | Distant lymph nodes | 6.11 |  | BRAF | V600E | **good** | CVac |
| 10 | Occult | F | 45 | III | Regional lymph nodes | 5.78 |  | BRAF | V600E | poor | VMCL vaccine # |
| 11 | Leg | M | 52 | IIIC | in-transit metastases | 2.20 |  | BRAF | V600E | poor | palliative radiotherapy |
| 12 | Leg | F | 39 | IV | Regional lymph nodes | 2.00 |  | NRAS | Q61K | **good** | palliative radiotherapy |
| 13 | Leg | F | 77 | IIIC | Regional lymph nodes | 1.41 |  | NRAS | Q61R | poor | - |
| 14 | Arm | M | 63 | IV | Regional lymph nodes | 0.89 |  | NRAS | Q61R | **good** | - |

**Supplementary Table S6:** Relative average expression ratios of PMEL, ICAM-1 and ITGAV normalized to the house keeping protein clathrin heavy chain (CV 17%) in the 32 fresh frozen AJCC stage III and IV metastatic melanoma specimens analysed by SWATH-MS and average FPKM normalized transcript levels of PMEL, ICAM-1 and ITGAV in 69 AJCC stage III metastatic melanoma in the TCGA-EE dataset.

|  | **SWATH-MS** | | **TCGA-EE dataset** | |
| --- | --- | --- | --- | --- |
| **Protein** | **high value**  **(fold lower than CLH1)** | **low value**  **(fold lower than CLH1)** | **high value (FPKM)** | **low value (FPKM)** |
| **PMEL** | 4.9 ± 3.2 | 33.8 ± 15.1 | 2959.6 ± 2613.3 | 39.2 ± 52.5 |
| **ICAM-1** | 8.1 ± 2.4 | 25.4 ± 10.5 | 90.6 ± 36 | 29.8 ± 12.5 |
| **ITGAV** | 32.6 ± 10.6 | 79.4 ± 31.6 | 23.9 ± 7.5 | 9.6 ± 3.6 |

**Supplementary Table S7:** Post-surgery relapse free survival analysis of disease free AJCC Stage III melanoma patients investigated by SWATH-MS grouped based on presence and absence of ICAM-1, PMEL and ITGAV.

| **Expression of Marker** | **p-value** | **Hazard ratio** | **95% CI** | **median relapse free survival**  **(months)** |
| --- | --- | --- | --- | --- |
| **ICAM-1 low**  **vs**  **ICAM-1 high** | < 0.0001 | 10.27 | 3.3 – 31.9 | 5.5  vs  64.0 |
| **ICAM-1 low, PMEL low**  **vs**  **ICAM-1 high, PMEL low** | < 0.0001 | 30.08 | 5.7 – 158.3 | 6.0  vs  109.2 |
| **ICAM-1 low, PMEL high, ITGAV high**  **vs**  **ICAM-1 low, PMEL high, ITGAV low** | 0.688 | 1.55 | 0.07 – 5.5 | 3.5  vs  5.0 |
| **ICAM-1 low, PMEL high, ITGAV high**  **vs**  **ICAM-1 high, PMEL high** | 0.186 | 4.90 | 0.5 – 51.9 | 3.5  vs  7.0 |
| **ICAM-1 low, PMEL high, ITGAV low**  **vs**  **ICAM-1 high, PMEL high** | 0.321 | 2.47 | 0.4 – 14.7 | 5.0  vs  7.0 |

**Supplementary Table S8:** Tumor pathology and patient survival of stage III specimens submitted to TCGA-SKCM dataset with submitter identifier TCGA-EE

| Sample ID | Gender | Age at diagnosis | Stage at diagnosis | NRAS/BRAF mutational status | Vital status | Months to last follow up from diagnosis | Months to death from diagnosis | Months to surgery (specimen submission) | Post-surgery survival (Months) | Tissue type collected |
| --- | --- | --- | --- | --- | --- | --- | --- | --- | --- | --- |
| TCGA-EE-A17X | male | 54 | Ia | NRAS mut | dead | n/a | 29.82 | 18.25 | 11.57 | Regional Lymph Node |
| TCGA-EE-A17Y | male | 69 | IIIb | BRAF mut | dead | n/a | 27.22 | 16.67 | 10.55 | Regional Cutaneous or Subcutaneous Tissue (includes satellite and in-transit metastasis) |
| TCGA-EE-A17Z | male | 57 | IIB | wt | dead | n/a | 8.64 | 2.33 | 6.31 | Regional Lymph Node |
| TCGA-EE-A181 | female | 82 | II | wt | dead | n/a | 33.71 | 32.59 | 1.12 | Regional Lymph Node |
| TCGA-EE-A183 | male | 48 | OCC | BRAF mut | dead | n/a | 26.89 | 14.70 | 12.20 | Regional Lymph Node |
| TCGA-EE-A185 | female | 55 | IIIc | NRAS mut | dead | n/a | 4.96 | 0.66 | 4.31 | Regional Lymph Node |
| TCGA-EE-A20B | female | 66 | II | BRAF mut | alive | 133.81 | n/a | 73.58 | 60.23 | Regional Cutaneous or Subcutaneous Tissue (includes satellite and in-transit metastasis) |
| TCGA-EE-A20F | male | 53 | I | BRAF mut | alive | 91.56 | n/a | 75.72 | 15.85 | Regional Lymph Node |
| TCGA-EE-A29C | male | 20 | Ib | BRAF mut | dead | 47.84 | 78.97 | 9.86 | 69.11 | Regional Lymph Node |
| TCGA-EE-A29D | male | 87 | IIIC | wt | dead | n/a | 13.96 | 2.83 | 11.14 | Regional Lymph Node |
| TCGA-EE-A29E | male | 54 | IIIb | BRAF mut | alive | 63.78 | n/a | 0.99 | 62.79 | Regional Lymph Node |
| TCGA-EE-A29G | male | 53 | IIIa | NRAS mut | dead | n/a | 72.07 | 63.52 | 8.55 | Regional Cutaneous or Subcutaneous Tissue (includes satellite and in-transit metastasis) |
| TCGA-EE-A29H | female | 59 | Ia | BRAF mut | alive | 64.64 | n/a | 27.78 | 36.85 | Regional Lymph Node |
| TCGA-EE-A29L | male | 78 | IIIc | NRAS mut | dead | n/a | 2.60 | 1.51 | 1.08 | Regional Lymph Node |
| TCGA-EE-A29M | female | 33 | Ib | BRAF mut | alive | 56.84 | n/a | 17.23 | 39.62 | Regional Lymph Node |
| TCGA-EE-A29N | male | 78 | I or II NOS | wt | dead | n/a | 18.60 | 9.10 | 9.49 | Regional Lymph Node |
| TCGA-EE-A29P | female | 73 | IIc | BRAF mut | alive | 56.42 | n/a | 44.38 | 12.03 | Regional Lymph Node |
| TCGA-EE-A29Q | female | 70 | IIb | NRAS mut | dead | 37.35 | 66.74 | 29.26 | 37.48 | Regional Cutaneous or Subcutaneous Tissue (includes satellite and in-transit metastasis) |
| TCGA-EE-A29R | female | 48 | IIIc | NRAS mut | alive | 14.47 | n/a | 5.62 | 8.84 | Regional Lymph Node |
| TCGA-EE-A29S | male | 79 | IIa | NRAS mut | dead | 55.92 | 61.28 | 45.93 | 15.35 | Regional Lymph Node |
| TCGA-EE-A29T | female | 51 | I - III NOS | BRAF mut | alive | 369.93 | n/a | 339.48 | 30.44 | Regional Lymph Node |
| TCGA-EE-A29W | male | 42 | OCC | BRAF mut | alive | 195.02 | n/a | 162.87 | 32.15 | Regional Lymph Node |
| TCGA-EE-A29X | female | 58 | Ib | NRAS mut | dead | n/a | 17.92 | 9.83 | 8.09 | Regional Lymph Node |
| TCGA-EE-A2A0 | female | 77 | IIA | wt | dead | n/a | 46.78 | 35.48 | 11.30 | Regional Lymph Node |
| TCGA-EE-A2A1 | male | 46 | Ib | NRAS mut | alive | 115.96 | n/a | 53.69 | 62.27 | Regional Lymph Node |
| TCGA-EE-A2A2 | male | 71 | IIIc | NRAS mut | alive | 59.64 | n/a | 0.53 | 59.11 | Regional Lymph Node |
| TCGA-EE-A2A5 | male | 43 | Ib | NRAS mut | dead | n/a | 39.29 | 29.62 | 9.67 | Regional Cutaneous or Subcutaneous Tissue (includes satellite and in-transit metastasis) |
| TCGA-EE-A2A6 | male | 43 | Ia | BRAF mut | alive | 86.14 | n/a | 49.81 | 36.33 | Regional Lymph Node |
| TCGA-EE-A2GB | male | 51 | IIIb | BRAF mut | alive | 59.28 | n/a | 8.15 | 51.12 | Regional Lymph Node |
| TCGA-EE-A2GC | male | 82 | IIB | wt | alive | 67.38 | n/a | 33.12 | 34.27 | Regional Lymph Node |
| TCGA-EE-A2GD | female | 58 | IIb | NRAS mut | dead | 314.56 | 340.14 | 296.58 | 43.56 | Regional Cutaneous or Subcutaneous Tissue (includes satellite and in-transit metastasis) |
| TCGA-EE-A2GE | male | 44 | I | BRAF mut | alive | 173.79 | n/a | 101.03 | 72.76 | Regional Lymph Node |
| TCGA-EE-A2GH | male | 34 | I | BRAF mut | alive | 220.24 | n/a | 160.83 | 59.41 | Regional Lymph Node |
| TCGA-EE-A2GI | male | 39 | IA | wt | alive | 48.69 | n/a | 18.63 | 30.06 | Regional Lymph Node |
| TCGA-EE-A2GJ | male | 83 | Ia | NRAS mut | dead | 89.33 | 107.38 | 69.90 | 37.48 | Regional Lymph Node |
| TCGA-EE-A2GK | female | 46 | I | wt | alive | 54.70 | n/a | 38.70 | 16.00 | Regional Lymph Node |
| TCGA-EE-A2GL | female | 40 | IIa | NRAS mut | alive | 79.66 | n/a | 44.12 | 35.54 | Regional Lymph Node |
| TCGA-EE-A2GM | female | 70 | IIc | NRAS mut | alive | 75.48 | n/a | 47.18 | 28.31 | Regional Cutaneous or Subcutaneous Tissue (includes satellite and in-transit metastasis) |
| TCGA-EE-A2GN | male | 67 | IIa | NRAS mut | dead | 90.97 | 102.12 | 57.14 | 44.98 | Regional Cutaneous or Subcutaneous Tissue (includes satellite and in-transit metastasis) |
| TCGA-EE-A2GO | female | 66 | II | NRAS mut | alive | 126.81 | n/a | 71.11 | 55.69 | Regional Cutaneous or Subcutaneous Tissue (includes satellite and in-transit metastasis) |
| TCGA-EE-A2GP | male | 80 | IIIb | NRAS mut | dead | n/a | 13.91 | 8.48 | 5.42 | Regional Cutaneous or Subcutaneous Tissue (includes satellite and in-transit metastasis) |
| TCGA-EE-A2GR | male | 78 | II | wt | dead | 14.29 | 42.74 | 13.14 | 29.60 | Regional Skin or Soft Tissue |
| TCGA-EE-A2GS | female | 28 | Ib | BRAF mut | dead | 55.59 | 81.21 | 42.58 | 38.63 | Regional Cutaneous or Subcutaneous Tissue (includes satellite and in-transit metastasis) |
| TCGA-EE-A2GT | male | 77 | IIa | BRAF mut | alive | 44.88 | n/a | 9.90 | 34.98 | Regional Lymph Node |
| TCGA-EE-A2GU | female | 65 | Ia | NRAS mut | alive | 94.82 | n/a | 58.49 | 36.33 | Regional Lymph Node |
| TCGA-EE-A2M5 | male | 49 | I | BRAF mut | dead | n/a | 21.67 | 12.82 | 8.84 | Regional Lymph Node |
| TCGA-EE-A2M6 | male | 61 | I | BRAF mut | alive | 129.27 | n/a | 31.00 | 98.27 | Regional Lymph Node |
| TCGA-EE-A2M7 | male | 66 | II | BRAF mut | dead | n/a | 28.83 | 26.14 | 2.70 | Regional Lymph Node |
| TCGA-EE-A2M8 | female | 54 | III | BRAF mut | dead | n/a | 19.76 | 13.58 | 6.18 | Regional Lymph Node |
| TCGA-EE-A2MC | male | 73 | I | NRAS mut | dead | n/a | 61.51 | 14.01 | 47.51 | Regional Lymph Node |
| TCGA-EE-A2MD | male | 52 | II | NRAS mut | dead | n/a | 47.28 | 37.74 | 9.53 | Regional Lymph Node |
| TCGA-EE-A2ME | male | 51 | I | BRAF mut | dead | n/a | 103.27 | 73.81 | 29.46 | Regional Lymph Node |
| TCGA-EE-A2MF | female | 39 | I | NRAS mut | dead | n/a | 268.73 | 266.01 | 2.73 | Regional Lymph Node |
| TCGA-EE-A2MG | male | 23 | I | BRAF mut | dead | n/a | 103.20 | 58.95 | 44.25 | Regional Lymph Node |
| TCGA-EE-A2MH | male | 66 | III | BRAF mut | dead | n/a | 16.96 | 9.01 | 7.96 | Regional Lymph Node |
| TCGA-EE-A2MI | male | 43 | I | wt | dead | n/a | 204.52 | 189.96 | 14.55 | Regional Lymph Node |
| TCGA-EE-A2MJ | male | 60 | IIB | wt | dead | n/a | 96.16 | 32.79 | 63.38 | Regional Lymph Node |
| TCGA-EE-A2MK | female | 18 | III | BRAF mut | alive | 180.39 | n/a | 32.98 | 147.42 | Regional Lymph Node |
| TCGA-EE-A2ML | male | 35 | II | NRAS mut | dead | 203.05 | 216.66 | 91.13 | 125.52 | Regional Lymph Node |
| TCGA-EE-A2MM | female | 63 | I | NRAS mut | dead | n/a | 167.90 | 111.88 | 56.02 | Regional Lymph Node |
| TCGA-EE-A2MN | male | 58 | I | NRAS mut | dead | n/a | 47.54 | 22.45 | 25.08 | Regional Lymph Node |
| TCGA-EE-A2MP | female | 34 | I | BRAF mut | alive | 248.65 | n/a | 221.65 | 26.99 | Regional Lymph Node |
| TCGA-EE-A2MQ | female | 70 | IIIa | BRAF mut | dead | n/a | 43.23 | 21.63 | 21.60 | Regional Lymph Node |
| TCGA-EE-A2MR | male | 61 | IB | wt | alive | 134.31 | n/a | 73.86 | 60.45 | Regional Lymph Node |
| TCGA-EE-A2MS | male | 72 | IB | wt | alive | 162.37 | n/a | 109.24 | 53.13 | Regional Lymph Node |
| TCGA-EE-A2MT | male | 45 | IB | wt | alive | 71.16 | n/a | 18.53 | 52.63 | Regional Lymph Node |
| TCGA-EE-A2MU | male | 71 | Ia | NRAS mut | alive | 53.26 | n/a | 47.70 | 5.56 | Regional Cutaneous or Subcutaneous Tissue (includes satellite and in-transit metastasis) |
| TCGA-EE-A3AA | male | 47 | III | BRAF mut | alive | 124.31 | n/a | 0.00 | 124.31 | Regional Lymph Node |
| TCGA-EE-A3AB | male | 30 | III | BRAF mut | alive | 122.73 | n/a | 0.00 | 122.73 | Regional Lymph Node |

**Supplementary Table S9**: Relative protein expression of MITF targeted genes in MEKi sensitive compared to MEKi insensitive melanoma cell lines.

| UniProt Accession | UniProt ID | Protein Name | Fold change (sen/insen) | Student’s T-Test | MEKi correlation |
| --- | --- | --- | --- | --- | --- |
| Q96LR9 | APLD1_HUMAN | Apolipoprotein L domain-containing protein 1 | *n/d* | *n/d* | *n/d* |
| Q13510 | ASAH1_HUMAN | Acid ceramidase | 2.81 | 5.2e^-10^ | -0.73 |
| P10415 | BCL2_HUMAN | Apoptosis regulator Bcl-2 | *n/d* | *n/d* | *n/d* |
| O76090 | BEST1_HUMAN | Bestrophin-1 | *n/d* | *n/d* | *n/d* |
| Q96CA5 | BIRC7_HUMAN | Baculoviral IAP repeat-containing protein 7 | *n/d* | *n/d* | *n/d* |
| P20807 | CAN3_HUMAN | Calpain-3 | *n/d* | *n/d* | *n/d* |
| P24941 | CDK2_HUMAN | Cyclin-dependent kinase 2 | 2.21 | 6.3e^-4^ | -0.72 |
| P51798 | CLCN7_HUMAN | H(+)/Cl(-) exchange transporter 7 | *n/d* | *n/d* | *n/d* |
| P24530 | EDNRB_HUMAN | Endothelin B receptor | *n/d* | *n/d* | *n/d* |
| Q6NXG1 | ESRP1_HUMAN | Epithelial splicing regulatory protein 1 | *n/d* | *n/d* | *n/d* |
| Q9Y2L6 | FRM4B_HUMAN | FERM domain-containing protein 4B | *n/d* | *n/d* | *n/d* |
| P36959 | GMPR1_HUMAN | GMP reductase 1 | *n/d* | *n/d* | *n/d* |
| P51810 | GP143_HUMAN | G-protein coupled receptor 143 | 8.65 | 7.2e^-6^ | -0.69 |
| Q14956 | GPNMB_HUMAN | Transmembrane glycoprotein NMB | 9.87 | 3.7e^-9^ | -0.71 |
| Q4ZG55 | GREB1_HUMAN | Protein GREB1 | *n/d* | *n/d* | *n/d* |
| O15327 | INP4B_HUMAN | Type II inositol 3,4-bisphosphate 4-phosphatase | *n/d* | *n/d* | *n/d* |
| Q15306 | IRF4_HUMAN | Interferon regulatory factor 4 | *n/d* | *n/d* | *n/d* |
| Q9H2S1 | KCNN2_HUMAN | Small conductance calcium-activated potassium channel protein 2 | *n/d* | *n/d* | *n/d* |
| P17931 | LEG3_HUMAN | Galectin-3 | 2.12 | 6.0e^-3^ | -0.61 |
| Q16655 | MAR1_HUMAN | Melanoma antigen recognized by T-cells 1 | 7.47 | 2.0e^-6^ | -0.76 |
| P02686 | MBP_HUMAN | Myelin basic protein | 0.57 | 9.2e^-3^ | 0.13 |
| Q8TDZ2 | MICA1_HUMAN | Protein-methionine sulfoxide oxidase MICAL1 | 0.65 | 9.1e^-2^ | 0.27 |
| Q86WC4 | OSTM1_HUMAN | Osteopetrosis-associated transmembrane protein 1 | *n/d* | *n/d* | *n/d* |
| Q53H76 | PLA1A_HUMAN | Phospholipase A1 member A | *n/d* | *n/d* | *n/d* |
| P40967 | PMEL_HUMAN | Melanocyte protein PMEL | 5.91 | 1.6e^-9^ | -0.92 |
| P13686 | PPA5_HUMAN | Tartrate-resistant acid phosphatase type 5 | 3.22 | 3.3e^-2^ | -0.28 |
| P51159 | RB27A_HUMAN | Ras-related protein Rab-27A | 3.35 | 2.3e^-4^ | -0.73 |
| Q9UMX9 | S45A2_HUMAN | Membrane-associated transporter protein | *n/d* | *n/d* | *n/d* |
| P43007 | SATT_HUMAN | Neutral amino acid transporter A | 1.13 | 7.2e^-1^ | -0.09 |
| Q8TBP0 | TBC16_HUMAN | TBC1 domain family member 16 | *n/d* | *n/d* | *n/d* |
| Q7Z403 | TMC6_HUMAN | Transmembrane channel-like protein 6 | *n/d* | *n/d* | *n/d* |
| Q92956 | TNR14_HUMAN | Tumor necrosis factor receptor superfamily member 14 | *n/d* | *n/d* | *n/d* |
| Q7Z4N2 | TRPM1_HUMAN | Transient receptor potential cation channel subfamily M member 1 | *n/d* | *n/d* | *n/d* |
| P14679 | TYRO_HUMAN | Tyrosinase | 2.01 | 9.6e^-4^ | -0.75 |
| P17643 | TYRP1_HUMAN | 5,6-dihydroxyindole-2-carboxylic acid oxidase | 2.95 | 3.1e^-3^ | -0.65 |
| P40126 | TYRP2_HUMAN | L-dopachrome tautomerase | 3.09 | 4.2e^-3^ | -0.59 |
